# Gradient-type penalty method with inertial effects for solving constrained convex optimization problems with smooth data

**DOI:** 10.1007/s11590-017-1158-1

**Published:** 2017-06-14

**Authors:** Radu Ioan Boţ, Ernö Robert Csetnek, Nimit Nimana

**Affiliations:** 1 Faculty of Mathematics, University of Vienna, Oskar-Morgenstern-Platz 1, 1090 Vienna, Austria; 2 Faculty of Mathematics and Computer Sciences, Babeş-Bolyai University, Str. M. Kogălniceanu nr. 1, 400084 Cluj-Napoca, Romania; 3 Department of Mathematics, Faculty of Science, Naresuan University, Phitsanulok, 65000 Thailand

**Keywords:** Gradient method, Penalization, Fenchel conjugate, Inertial algorithm

## Abstract

We consider the problem of minimizing a smooth convex objective function subject to the set of minima of another differentiable convex function. In order to solve this problem, we propose an algorithm which combines the gradient method with a penalization technique. Moreover, we insert in our algorithm an inertial term, which is able to take advantage of the history of the iterates. We show weak convergence of the generated sequence of iterates to an optimal solution of the optimization problem, provided a condition expressed via the Fenchel conjugate of the constraint function is fulfilled. We also prove convergence for the objective function values to the optimal objective value. The convergence analysis carried out in this paper relies on the celebrated Opial Lemma and generalized Fejér monotonicity techniques. We illustrate the functionality of the method via a numerical experiment addressing image classification via support vector machines.

## Introduction and preliminaries

Let *H* be a real Hilbert space with the norm and inner product given by $\Vert \cdot \Vert$ and $\langle \cdot ,\cdot \rangle$, respectively, and *f* and *g* be convex functions acting on *H*, which we assume for simplicity to be everywhere defined and (Fréchet) differentiable. The object of our investigation is the optimization problem

1 $$\begin{aligned} \min _{x\in \mathrm{argmin}\,g} f(x). \end{aligned}$$

We assume that

$$\begin{aligned} \mathcal {S}:=\mathrm{argmin}\,\left\{ f(x):x\in \mathrm{argmin}\,g\right\} \ne \emptyset \end{aligned}$$

and that the gradients $\nabla f$ and $\nabla g$ are Lipschitz continuous operators with constants $L_f$ and $L_g$, respectively.

The work [5] of Attouch and Czarnecki has attracted since its appearance a huge interest from the research community, since it undertakes a qualitative analysis of the optimal solutions of (1) from the perspective of a penalty-term based dynamical system. This represented the starting point for the design and development of numerical algorithms for solving the minimization problem (1), several variants of it involving also nonsmooth data up to monotone inclusions that are related to optimality systems of constrained optimization problems. We refer the reader to [4–8, 10, 11, 13–15, 20–23, 33, 35] and the references therein for more insights into this research topic.

A key assumption used in this context in order to guarantee the convergence properties of the numerical algorithms is the condition

$$\begin{aligned} \sum _{n=1}^\infty \lambda _n\beta _n\left[ g^*\left( \frac{p}{\beta _n}\right) -\sigma _{\mathrm{argmin}\,g}\left( \frac{p}{\beta _n}\right) \right] <+\infty \quad \forall p\in \mathrm {ran}(N_{\mathrm{argmin}\,g}), \end{aligned}$$

where $\{\lambda _n\}_{n=1}^\infty$ and $\{\beta _n\}_{n=1}^\infty$ are positive sequences, $g^*: {{\mathcal {H}}}\rightarrow \mathbb {R}\cup \{+\infty \}$ is the Fenchel conjugate of *g*:

$$\begin{aligned} g^*(p)=\sup _{x\in {{\mathcal {H}}}}\{\langle p,x\rangle -g(x)\} \quad \forall p\in {{\mathcal {H}}}; \end{aligned}$$

$\sigma _{\mathrm{argmin}\,g}: {{\mathcal {H}}}\rightarrow \mathbb {R}\cup \{+\infty \}$ is the support function of the set $\mathrm{argmin}\,g$:

$$\begin{aligned} \sigma _{\mathrm{argmin}\,g}(p)=\sup _{x\in {\mathrm{argmin}\,g}}\langle p,x\rangle \quad \forall p\in {{\mathcal {H}}}; \end{aligned}$$

and $N_{\mathrm{argmin}\,g}$ is the normal cone to the set $\mathrm{argmin}\,g$, defined by

$$\begin{aligned} N_{\mathrm{argmin}\,g}(x)=\{p\in {{\mathcal {H}}}:\langle p,y-x\rangle \le 0 \ \forall y\in \mathrm{argmin}\,g\} \end{aligned}$$

for $x \in \mathrm{argmin}\,g$ and $N_{\mathrm{argmin}\,g}(x)=\emptyset$ for $x\not \in \mathrm{argmin}\,g$. Finally, $\mathrm{ran}\,(N_{\mathrm{argmin}\,g})$ denotes the range of the normal cone $N_{\mathrm{argmin}\,g}$, that is, $p\in \mathrm{ran}\,(N_{\mathrm{argmin}\,g})$ if and only if there exists $x\in \mathrm{argmin}\,g$ such that $p\in N_{\mathrm{argmin}\,g}(x)$. Let us notice that for $x\in {\mathrm{argmin}\,g}$ one has $p\in N_{\mathrm{argmin}\,g}(x)$ if and only if $\sigma _{\mathrm{argmin}\,g}(p)=\langle p,x\rangle$. We also assume without loss of generality that $\min g=0$.

In this paper we propose a numerical algorithm for solving (1) that combines the gradient method with penalization strategies also by employing inertial and memory effects. Algorithms of inertial type result from the time discretization of differential inclusions of second order type (see [1, 3]) and were first investigated in the context of the minimization of a differentiable function by Polyak [36] and Bertsekas [12]. The resulting iterative schemes share the feature that the next iterate is defined by means of the last two iterates, a fact which induces the inertial effect in the algorithm. Since the works [1, 3], one can notice an increasing number of research efforts dedicated to algorithms of inertial type (see [1–3, 9, 16–19, 24–28, 30–32, 34]).

In this paper we consider the following inertial algorithm for solving (1):

### Algorithm 1

Initialization: Choose the positive sequences $\{\lambda _n\}_{n=1}^\infty$ and $\{\beta _n\}_{n=1}^\infty$, and a positive constant parameter $\alpha \in (0,1)$. Take arbitrary $x_0,x_1\in H$.

Iterative step: For given current iterates $x_{n-1}, x_n\in H$ ($n\ge 1$), define $x_{n+1}\in H$ by

$$\begin{aligned} x_{n+1}:=x_n+\alpha (x_n-x_{n-1})-\lambda _n\nabla f(x_n) - \lambda _n\beta _n \nabla g(x_n). \end{aligned}$$

We notice that in the above iterative scheme $\{\lambda _n\}_{n=1}^\infty$ represents the sequence of step sizes, $\{\beta _n\}_{n=1}^\infty$ the sequence of penalty parameters, while $\alpha$ controls the influence of the inertial term.

For every $n \ge 1$ we denote by $\Omega _n:=f+\beta _n g$, which is also a (Fréchet) differentiable function, and notice that $\nabla \Omega _n$ is $L_n:= L_f+\beta _n L_g$-Lipschitz continuous.

In case $\alpha =0$, Algorithm 1 collapses in the algorithm considered in [35] for solving (1). We prove weak convergence for the generated iterates to an optimal solution of (1), by making use of generalized Fejér monotonicity techniques and the Opial Lemma and by imposing the key assumption mentioned above as well as some mild conditions on the involved parameters. Moreover, the performed analysis allows us also to show the convergence of the objective function values to the optimal objective value of (1). As an illustration of the theoretical results, we present in the last section an application addressing image classification via support vector machines.

## Convergence analysis

This section is devoted to the asymptotic analysis of Algorithm 1.

### Assumption 2

Assume that the following statements hold:

(I) The function *f* is bounded from below;
(II) There exist positive constants $c>1$ and $K>0$ such that $\frac{L_n}{2}+\frac{\alpha -1}{\lambda _n}\le -\left( c+(1+\alpha )K\right)$ and $\beta _{n+1}-\beta _n\le K\lambda _{n+1}\beta _{n+1}$ for all $n\ge 1$;
(III) For every $p\in \mathrm {ran}(N_{\mathrm{argmin}\,g})$, we have $\sum _{n=1}^\infty \lambda _n\beta _n\left[ g^*\left( \frac{p}{\beta _n}\right) -\sigma _{\mathrm{argmin}\,g}\left( \frac{p}{\beta _n}\right) \right] <+\infty$;
(IV) $\liminf _{n\rightarrow +\infty }\lambda _n\beta _n>0$, $\left( \frac{1}{\lambda _{n+1}}-\frac{1}{\lambda _n}\right) \le \frac{2}{\alpha }$ for all $n \ge 1$ and $\sum _{n=1}^\infty \lambda _n=+\infty$.

We would like to mention that in [21] we proposed a forward-backward-forward algorithm of penalty-type, endowed with inertial and memory effects, for solving monotone inclusion problems, which gave rise to a primal-dual iterative scheme for solving convex optimization problems with complex structures. However, we succeeded in proving only weak ergodic convergence for the generated iterates, while with the specific choice of the sequences $\{\lambda _n\}_{n=1}^\infty$ and $\{\beta _n\}_{n=1}^\infty$ in Assumption 2 we will be able to prove weak convergence of the iterates generated in Algorithm 1 to an optimal solution of (1).

### Remark 3

The conditions in Assumption 2 slightly extend the ones considered in [35] in the noninertial case. The only differences are given by the first inequality in (II), which here involves the constant $\alpha$ which controls the inertial terms (for the corresponding condition in [35] one only has to take $\alpha =0$), and by the inequality $\left( \frac{1}{\lambda _{n+1}}-\frac{1}{\lambda _n}\right) \le \frac{2}{\alpha }$ for all $n \ge 1$.

We refer to Remark 12 for situations where the fulfillment of the conditions in Assumption 2 is guaranteed.

We start the convergence analysis with three technical results.

### Lemma 4

Let $\overline{x}\in \mathcal {S}$ and set $\overline{p}:=-\nabla f(\overline{x})$. We have for all $n \ge 1$

2 $$\begin{aligned}&\varphi _{n+1}-\varphi _n-\alpha \left( \varphi _n-\varphi _{n-1}\right) +\lambda _n\beta _ng(x_n)\nonumber \\&\quad \le \Vert x_{n+1}-x_n\Vert ^2+\alpha \Vert x_n-x_{n-1}\Vert ^2\nonumber \\&\qquad +\lambda _n\beta _n\left[ g^*\left( \frac{2\overline{p}}{\beta _n}\right) -\sigma _{\mathrm{argmin}\,g}\left( \frac{2\overline{p}}{\beta _n}\right) \right] , \end{aligned}$$

where $$\varphi _n:=\Vert x_n-\overline{x}\Vert ^2$$.

### Proof

Since $\overline{x}\in \mathcal {S}$, we have according to the first-order optimality conditions that $0\in \nabla f(\overline{x})+N_{\mathrm{argmin}\,g}(\overline{x})$, thus $\overline{p}=-\nabla f(\overline{x})\in N_{\mathrm{argmin}\,g}(\overline{x})$. Notice that for all $n \ge 1$

$$\begin{aligned} \nabla f(x_n)=\frac{y_n-x_{n+1}}{\lambda _n}-\beta _n\nabla g(x_n), \end{aligned}$$

where $y_n:=x_n+\alpha (x_n-x_{n-1})$. This, together with the monotonicity of $\nabla f$, imply that

3 $$\begin{aligned}&\left\langle \frac{y_n-x_{n+1}}{\lambda _n}-\beta _n\nabla g(x_n)+\overline{p},x_n-\overline{x}\right\rangle \nonumber \\&\qquad =\left\langle \nabla f(x_n)-\nabla f(\overline{x}),x_n-\overline{x}\right\rangle \ge 0 \quad \forall n \ge 1, \end{aligned}$$

so

4 $$\begin{aligned} 2\left\langle y_n-x_{n+1},x_n-\overline{x}\right\rangle \ge 2\lambda _n\beta _n\langle \nabla g(x_n),x_n-\overline{x}\rangle -2\lambda _n\langle \overline{p},x_n-\overline{x}\rangle \quad \forall n \ge 1.\quad \end{aligned}$$

On the other hand, since *g* is convex and differentiable, we have for all $n \ge 1$

$$\begin{aligned} 0=g(\overline{x})\ge g(x_n)+\langle \nabla g(x_n),\overline{x}-x_n\rangle , \end{aligned}$$

which means that

5 $$\begin{aligned} 2\lambda _n\beta _n\langle \nabla g(x_n),x_n-\overline{x}\rangle \ge 2\lambda _n\beta _ng(x_n). \end{aligned}$$

As for all $n \ge 1$

$$\begin{aligned} 2\langle x_n-x_{n+1},x_n-\overline{x}\rangle =\Vert x_{n+1}-x_n\Vert ^2+\varphi _n-\varphi _{n+1} \end{aligned}$$

and

$$\begin{aligned} 2\alpha \langle x_n-x_{n-1},x_n-\overline{x}\rangle =\alpha \Vert x_{n}-x_{n-1}\Vert ^2+\alpha \left( \varphi _n-\varphi _{n-1}\right) , \end{aligned}$$

it follows

6 $$\begin{aligned} 2\langle y_n-x_{n+1},x_n-\overline{x}\rangle= & {} 2\langle x_n-x_{n+1},x_n-\overline{x}\rangle +2\alpha \langle x_n-x_{n-1},x_n-\overline{x}\rangle \nonumber \\= & {} \Vert x_{n+1}-x_n\Vert ^2+\alpha \Vert x_{n}-x_{n-1}\Vert ^2\nonumber \\&+\,\varphi _n-\varphi _{n+1}+\alpha \left( \varphi _n-\varphi _{n-1}\right) . \end{aligned}$$

Combining (4), (5) and (6), we obtain that for each $n\ge 1$

7 $$\begin{aligned}&\varphi _{n+1}-\varphi _n- \alpha \left( \varphi _n-\varphi _{n-1}\right) +\lambda _n\beta _ng(x_n) \nonumber \\&\quad \le \Vert x_{n+1}-x_n\Vert ^2+\alpha \Vert x_{n}-x_{n-1}\Vert ^2 -\lambda _n\beta _ng(x_n)\nonumber \\&\qquad +\,2\lambda _n\langle \overline{p},x_n\rangle -2\lambda _n\langle \overline{p}, \overline{x}\rangle . \end{aligned}$$

Finally, since $\overline{x}\in \mathrm{argmin}\,g$, we have that for all $n\ge 1$

$$\begin{aligned} 2\lambda _n\langle \overline{p}, x_n\rangle -\lambda _n\beta _n g(x_n)-2\lambda _n\langle \overline{p},\overline{x}\rangle= & {} \lambda _n\beta _n\left[ \left\langle \frac{2\overline{p}}{\beta _n},x_n\right\rangle -g(x_n)-\left\langle \frac{2\overline{p}}{\beta _n},\overline{x}\right\rangle \right] \\\le & {} \lambda _n\beta _n\left[ g^*\left( \frac{2\overline{p}}{\beta _n}\right) -\left\langle \frac{2\overline{p}}{\beta _n},\overline{x}\right\rangle \right] \\= & {} \lambda _n\beta _n\left[ g^*\left( \frac{2\overline{p}}{\beta _n}\right) -\sigma _{\mathrm{argmin}\,g}\left( \frac{2\overline{p}}{\beta _n}\right) \right] , \end{aligned}$$

which completes the proof. $\square$

### Lemma 5

We have for all $n \ge 1$

8 $$\begin{aligned} \Omega _{n+1}(x_{n+1})\le & {} \Omega _n(x_n)+(\beta _{n+1}-\beta _n)g(x_{n+1})\nonumber \\&+\left[ \frac{L_n}{2}+\frac{\alpha }{2\lambda _n}-\frac{1}{\lambda _n}\right] \Vert x_{n+1}-x_n\Vert ^2+\frac{\alpha }{2\lambda _n}\Vert x_n-x_{n-1}\Vert ^2. \end{aligned}$$

### Proof

From the descent Lemma and the fact that $\nabla \Omega _n$ is $L_n$-Lipschitz continuous, we get that

$$\begin{aligned} \Omega _{n}(x_{n+1})\le \Omega _n(x_n)+\left\langle \nabla \Omega _n(x_n),x_{n+1}-x_n\right\rangle +\frac{L_n}{2}\Vert x_{n+1}-x_n\Vert ^2 \quad \forall n \ge 1. \end{aligned}$$

Since $\nabla \Omega _n(x_n)=-\frac{x_{n+1}-y_n}{\lambda _n}$, it holds for all $n \ge 1$

$$\begin{aligned} f(x_{n+1})+\beta _ng(x_{n+1})\le & {} f(x_n)+\beta _ng(x_n)\\&-\left\langle \frac{x_{n+1}-y_n}{\lambda _n},x_{n+1}-x_n\right\rangle +\frac{L_n}{2}\Vert x_{n+1}-x_n\Vert ^2 \end{aligned}$$

and then

$$\begin{aligned} f(x_{n+1})+\beta _{n+1}g(x_{n+1})\le & {} f(x_n)+\beta _ng(x_n)+(\beta _{n+1}-\beta _n)g(x_{n+1})\\&-\frac{1}{\lambda _n}\Vert x_{n+1}-x_n\Vert ^2+\frac{\alpha }{\lambda _n}\left\langle x_{n}-x_{n-1},x_{n+1}-x_n\right\rangle \\&+\frac{L_n}{2}\Vert x_{n+1}-x_n\Vert ^2, \end{aligned}$$

which is nothing else than

9 $$\begin{aligned} \Omega _{n+1}(x_{n+1})\le & {} \Omega _n(x_n)+(\beta _{n+1}-\beta _n)g(x_{n+1})+\left[ \frac{L_n}{2}-\frac{1}{\lambda _n}\right] \Vert x_{n+1}-x_n\Vert ^2\nonumber \\&+\frac{\alpha }{\lambda _n}\left\langle x_{n}-x_{n-1},x_{n+1}-x_n\right\rangle . \end{aligned}$$

By the Cauchy–Schwarz inequalty it holds that

$$\begin{aligned} \langle x_n-x_{n-1},x_{n+1}-x_n\rangle\le & {} \frac{1}{2}\Vert x_{n-1}-x_n\Vert ^2+\frac{1}{2}\Vert x_{n+1}-x_n\Vert ^2, \end{aligned}$$

hence, (9) becomes

$$\begin{aligned} \Omega _{n+1}(x_{n+1})\le & {} \Omega _n(x_n)+(\beta _{n+1}-\beta _n)g(x_{n+1})+\frac{\alpha }{2\lambda _n}\Vert x_{n-1}-x_n\Vert ^2\nonumber \\&+\left[ \frac{L_n}{2}-\frac{1}{\lambda _n}+\frac{\alpha }{2\lambda _n}\right] \Vert x_{n+1}-x_n\Vert ^2 \quad \forall n \ge 1. \end{aligned}$$

For $\overline{x}\in \mathcal {S}$ and all $n\ge 1$, we set

$$\begin{aligned} \Gamma _n:= & {} f(x_n)+(1-K\lambda _n)\beta _ng(x_n)+K\varphi _n\\= & {} \Omega _n(x_n)-K\lambda _n\beta _ng(x_n)+K\varphi _n, \end{aligned}$$

and, for simplicity, we denote

$$\begin{aligned} \delta _n:= \left( \frac{1}{2\lambda _{n}}+K\right) \alpha +c. \end{aligned}$$

### Lemma 6

Let $\overline{x}\in \mathcal {S}$ and set $\overline{p}:=-\nabla f(\overline{x})$. We have for all $n \ge 2$

10 $$\begin{aligned} \Gamma _{n+1}-\Gamma _n-\alpha (\Gamma _n-\Gamma _{n-1})\le & {} -\delta _n\Vert x_{n+1}-x_n\Vert ^2+\alpha \left( \frac{1}{2\lambda _n}+K\right) \Vert x_n-x_{n-1}\Vert ^2\nonumber \\&+\,K\lambda _n\beta _n\left[ g^*\left( \frac{2\overline{p}}{\beta _n}\right) -\sigma _{\mathrm{argmin}\,g}\left( \frac{2\overline{p}}{\beta _n}\right) \right] \nonumber \\&+\,\alpha \left( \Omega _{n-1}(x_{n-1})-\Omega _n(x_n)\right) \nonumber \\&+\,\alpha K\left( \lambda _n\beta _ng(x_n)-\lambda _{n-1}\beta _{n-1}g(x_{n-1})\right) . \end{aligned}$$

### Proof

According to Lemma 5 and Assumption 2(II), (8) becomes for all $n \ge 1$

11 $$\begin{aligned} \Omega _{n+1}(x_{n+1})- \Omega _n(x_n)-K\lambda _{n+1}\beta _{n+1}g(x_{n+1})\le & {} -(K+\delta _n)\Vert x_{n+1}-x_n\Vert ^2\nonumber \\&+\frac{\alpha }{2\lambda _n}\Vert x_n-x_{n-1}\Vert ^2. \end{aligned}$$

On the other hand, after multiplying (2) by *K*, we obtain for all $n \ge 1$

12 $$\begin{aligned}&K\varphi _{n+1}-K\varphi _n - \alpha \left( K\varphi _n-K\varphi _{n-1}\right) +K\lambda _n\beta _ng(x_n)\nonumber \\&\quad \le K\Vert x_{n+1}-x_n\Vert ^2+K\alpha \Vert x_n-x_{n-1}\Vert ^2 + K\lambda _n\beta _n\left[ g^*\left( \frac{2\overline{p}}{\beta _n}\right) -\sigma _{\mathrm{argmin}\,g}\left( \frac{2\overline{p}}{\beta _n}\right) \right] . \end{aligned}$$

After summing up the relations (11) and (12) and adding on both sides of the resulting inequality the expressions $\alpha \left( \Omega _{n-1}(x_{n-1})-\Omega _n(x_n)\right)$ and $\alpha (K\lambda _n\beta _ng(x_n)-K\lambda _{n-1}\beta _{n-1}g(x_{n-1}))$ for all $n \ge 2$, we obtain the required statement. $\square$

The following proposition will play an essential role in the convergence analysis (see also [1–3, 16]).

### Proposition 7

Let $\{a_n\}_{n=1}^\infty , \{b_n\}_{n=1}^\infty$ and $\{c_n\}_{n=1}^\infty$ be real sequences and $\alpha \in [0,1)$ be given. Assume that $\{a_n\}_{n=1}^\infty$ is bounded from below, $\{b_n\}_{n=1}^\infty$ is nonnegative and $\sum _{n=1}^\infty c_n<+\infty$ such that

$$\begin{aligned} a_{n+1}-a_n-\alpha (a_n-a_{n-1})+b_n\le c_n \quad \forall n \ge 1. \end{aligned}$$

Then the following statements hold:

(i) $$\sum _{n=1}^\infty [a_n-a_{n-1}]_+<+\infty $$, where $$[t]_+:=\max \{t,0\}$$;
(ii) $\{a_n\}_{n=1}^\infty$ converges and $\sum _{n=1}^\infty b_n<+\infty$.

The following lemma collects some convergence properties of the sequences involved in our analysis.

### Lemma 8

Let $\overline{x}\in \mathcal {S}$. Then the following statements are true:

(i) The sequence $\{\Gamma _n\}_{n=1}^\infty$ is bounded from below.
(ii) $\sum _{n=1}^\infty \Vert x_{n+1}-x_n\Vert ^2<+\infty$ and $\lim _{n\rightarrow +\infty }\Gamma _n$ exists.
(iii) $\lim _{n\rightarrow +\infty }\Vert x_n-\overline{x}\Vert$ exists and $\sum _{n=1}^\infty \lambda _n\beta _ng(x_n)<+\infty$.
(iv) $$\lim _{n\rightarrow +\infty }\Omega _n(x_n)$$ exists.
(v) $\lim _{n\rightarrow +\infty }g(x_n)=0$ and every sequential weak cluster point of the sequence $\{x_n\}_{n=1}^\infty$ lies in $\mathrm{argmin}\,g$.

### Proof

We set $\overline{p}:=-\nabla f(\overline{x})$ and recall that $g(\overline{x}) = \min g =0$.

(i) Since *f* is convex and differentiable, it holds for all $n \ge 1$

$$\begin{aligned} \Gamma _n= & {} f(x_n)+(1-K\lambda _n)\beta _ng(x_n)+K\varphi _n\\\ge & {} f(x_n)+K\Vert x_n-\overline{x}\Vert ^2\\\ge & {} f(\overline{x})+\langle \nabla f(\overline{x}), x_n-\overline{x}\rangle +K\Vert x_n-\overline{x}\Vert ^2 \ge f(\overline{x})-\frac{\Vert \overline{p}\Vert ^2}{4K}, \end{aligned}$$

which means that $\{\Gamma _n\}_{n=1}^\infty$ is bounded from below. Notice that the first inequality in the above relation is a consequence of Assumption 2(II), since $\frac{1-\alpha }{\lambda _n}\ge c+(1+\alpha )K\ge K$, thus $\lambda _n K\le 1-\alpha \le 1$ for all $n\ge 1$.

(ii) For all $n\ge 2$, we may set

$$\begin{aligned} \mu _n:=\Gamma _{n}-\alpha \Gamma _{n-1}+\alpha \left( \frac{1}{2\lambda _{n}}+K\right) \Vert x_{n}-x_{n-1}\Vert ^2 \end{aligned}$$

and

$$\begin{aligned} u_n:=\Omega _{n-1}(x_{n-1})-\Omega _n(x_n)+K\lambda _n\beta _ng(x_n)-K\lambda _{n-1}\beta _{n-1}g(x_{n-1}). \end{aligned}$$

We fix a natural number $N_0\ge 2$. Then

$$\begin{aligned} \sum _{n=2}^{N_0} u_n=f(x_1)+(1-K\lambda _1)\beta _1g(x_1)-f(x_{N_0})-(1-K\lambda _{N_0})\beta _{N_0}g(x_{N_0}). \end{aligned}$$

Since *f* is bounded from below and $g(x_{N_0})\ge g(\overline{x})=0$, it follows that $\sum _{n=2}^\infty u_n<+\infty$.

We notice that $-\delta _n+\alpha \left( \frac{1}{2\lambda _{n+1}}+K\right) =\frac{\alpha }{2}\left( \frac{1}{\lambda _{n+1}}-\frac{1}{\lambda _n}\right) -c$ and, since $\left( \frac{1}{\lambda _{n+1}}-\frac{1}{\lambda _n}\right) \le \frac{2}{\alpha }$, we have for all $n\ge 1$

13 $$\begin{aligned} -\delta _n+\alpha \left( \frac{1}{2\lambda _{n+1}}+K\right) \le 1-c. \end{aligned}$$

Thus, according Lemma 6, we get for all $n\ge 2$

$$\begin{aligned} \mu _{n+1}-\mu _n= & {} \Gamma _{n+1}-\Gamma _n-\alpha (\Gamma _n-\Gamma _{n-1})+\alpha \left( \frac{1}{2\lambda _{n+1}}+K\right) \Vert x_{n+1}-x_n\Vert ^2\\&-\alpha \left( \frac{1}{2\lambda _n}+K\right) \Vert x_n-x_{n-1}\Vert ^2\\\le & {} -\delta _n\Vert x_{n+1}-x_n\Vert ^2+K\lambda _n\beta _n\left[ g^*\left( \frac{2\overline{p}}{\beta _n}\right) -\sigma _{\mathrm{argmin}\,g}\left( \frac{2\overline{p}}{\beta _n}\right) \right] \\&+\alpha u_n+\alpha \left( \frac{1}{2\lambda _{n+1}}+K\right) \Vert x_{n+1}-x_n\Vert ^2\\\le & {} (1-c)\Vert x_{n+1}-x_n\Vert ^2+K\lambda _n\beta _n\left[ g^*\left( \frac{2\overline{p}}{\beta _n}\right) -\sigma _{\mathrm{argmin}\,g}\left( \frac{2\overline{p}}{\beta _n}\right) \right] +\alpha u_n. \end{aligned}$$

We fix another natural number $N_1\ge 2$ and sum up the last inequality for $n=2,\ldots , N_1$. We obtain

14 $$\begin{aligned} \mu _{N_1+1}-\mu _{2}\le & {} (1-c)\sum _{n=2}^{N_1}\Vert x_{n+1}-x_n\Vert ^2\nonumber \\&+K\sum _{n=2}^{N_1}\lambda _n\beta _n\left[ g^*\left( \frac{2\overline{p}}{\beta _n}\right) -\sigma _{\mathrm{argmin}\,g}\left( \frac{2\overline{p}}{\beta _n}\right) \right] \nonumber \\&+\,\alpha \sum _{n=2}^{N_1}u_n, \end{aligned}$$

which, by taking into account Assumption 2(III), means that $\{\mu _n\}_{n=2}^\infty$ is bounded from above by a positive number that we denote by *M*. Consequently, for all $n \ge 2$ we have

$$\begin{aligned} \Gamma _{n+1}-\alpha \Gamma _n\le \mu _{n+1}\le M, \end{aligned}$$

so

$$\begin{aligned} \Gamma _{n+1}\le \alpha \Gamma _n+M, \end{aligned}$$

which further implies that

$$\begin{aligned} \Gamma _n\le \alpha ^{n-2}\Gamma _{2}+M\sum _{k=1}^{n-2}\alpha ^{k-1}\le \alpha ^{n-2}\Gamma _{2}+\frac{M}{1-\alpha } \quad \forall n \ge 3. \end{aligned}$$

We have for all $n \ge 2$

$$\begin{aligned} \mu _{n+1}\ge f(\overline{x})-\frac{\Vert \overline{p}\Vert ^2}{4K}-\alpha \Gamma _n, \end{aligned}$$

hence

15 $$\begin{aligned} -\mu _{n+1}\le \alpha \Gamma _{n}-f(\overline{x})+\frac{\Vert \overline{p}\Vert ^2}{4K}\le \alpha ^{n-1}\Gamma _{2}+\frac{\alpha M}{1-\alpha }-f(\overline{x})+\frac{\Vert \overline{p}\Vert ^2}{4K}. \end{aligned}$$

Consequently, for the arbitrarily chosen natural number $N_1\ge 2$, we have [see (14)]

$$\begin{aligned} (c-1)\sum _{n=2}^{N_1}\Vert x_{n+1}-x_n\Vert ^2\le & {} -\mu _{N_1+1}+\mu _{2}\\&+K\sum _{n=2}^{N_1}\lambda _n\beta _n\left[ g^*\left( \frac{2\overline{p}}{\beta _n}\right) -\sigma _{\mathrm{argmin}\,g}\left( \frac{2\overline{p}}{\beta _n}\right) \right] {+}\alpha \sum _{n=2}^{N_1}u_n, \end{aligned}$$

which together with (15) and the fact that $c>1$ implies that

$$\begin{aligned} \sum _{n=1}^\infty \Vert x_{n+1}-x_n\Vert ^2<+\infty . \end{aligned}$$

On the other hand, due to (13) we have $\delta _{n+1}\le \delta _n+1$ for all $n \ge 1$. Consequently, using also that $c >1$, (10) implies that

$$\begin{aligned} \Gamma _{n+1}-\Gamma _n-\alpha (\Gamma _n-\Gamma _{n-1})\le & {} -\delta _n\Vert x_{n+1}-x_n\Vert ^2+(\delta _{n}-c)\Vert x_n-x_{n-1}\Vert ^2\nonumber \\&+K\lambda _n\beta _n\left[ g^*\left( \frac{2\overline{p}}{\beta _n}\right) -\sigma _{\mathrm{argmin}\,g}\left( \frac{2\overline{p}}{\beta _n}\right) \right] +\alpha u_n\nonumber \\\le & {} -\delta _n\Vert x_{n+1}-x_n\Vert ^2+\delta _{n-1}\Vert x_n-x_{n-1}\Vert ^2\nonumber \\&+K\lambda _n\beta _n\left[ g^*\left( \frac{2\overline{p}}{\beta _n}\right) -\sigma _{\mathrm{argmin}\,g}\left( \frac{2\overline{p}}{\beta _n}\right) \right] +\alpha u_n \quad \forall n \ge 1. \end{aligned}$$

According to Proposition 7 and by taking into account that $\{\Gamma _n\}_{n=1}^\infty$ is bounded from below, we obtain that $\lim _{n\rightarrow +\infty }\Gamma _n$ exists.

(iii) By Lemma 4 and Proposition 7, $\lim _{n\rightarrow +\infty }\varphi _n$ exists and $\sum _{n=1}^\infty \lambda _n\beta _ng(x_n)<+\infty$.

(iv) Since $\Omega _n(x_n)=\Gamma _n-K\varphi _n+K\lambda _n\beta _ng(x_n)$ for all $n \ge 1$, by using (ii) and (iii), we get that $\lim _{n\rightarrow +\infty }\Omega _n(x_n)$ exists.

(v) Since $\liminf _{n\rightarrow +\infty }\lambda _n\beta _n>0$, we also obtain that $\lim _{n\rightarrow +\infty }g(x_n)=0.$ Let *w* be a sequential weak cluster point of $\{x_n\}_{n=1}^\infty$ and assume that the subsequence $\{x_{n_j}\}_{j=1}^\infty$ converges weakly to *w*. Since *g* is weak lower semicontinuous, we have

$$\begin{aligned} g(w)\le \liminf _{j\rightarrow +\infty }g(x_{n_j})=\lim _{n\rightarrow +\infty }g(x_n)=0, \end{aligned}$$

which implies that $w\in \mathrm{argmin}\,g$. This completes the proof. $\square$

In order to show also the convergence of the sequence $(f(x_n))_{n=1}^\infty$, we prove first the following result.

### Lemma 9

Let $\overline{x}\in \mathcal {S}$ be given. We have

$$\begin{aligned} \sum _{n=1}^\infty \lambda _n\left[ \Omega _n(x_n)-f(\overline{x})\right] <+\infty . \end{aligned}$$

### Proof

Since *f* is convex and differentiable, we have for all $n \ge 1$

$$\begin{aligned} f(\overline{x})\ge f(x_n)+\langle \nabla f(x_n),\overline{x}-x_n\rangle . \end{aligned}$$

Since *g* is convex and differentiable, we have for all $n \ge 1$

$$\begin{aligned} 0\ge \beta _ng(x_n)+\langle \beta _n\nabla g(x_n),\overline{x}-x_n\rangle , \end{aligned}$$

which together imply that

$$\begin{aligned} f(\overline{x})\ge & {} \Omega _n(x_n)+\langle \nabla \Omega _n(x_n),\overline{x}-x_n\rangle \\= & {} \Omega _n(x_n)+\left\langle \frac{y_n-x_{n+1}}{\lambda _n},\overline{x}-x_n\right\rangle \quad \forall n \ge 1. \end{aligned}$$

From here we obtain for all $n \ge 1$ [see (6)]

$$\begin{aligned} 2\lambda _n\left[ \Omega _n(x_n)-f(\overline{x})\right]\le & {} 2\langle y_n-x_{n+1},x_n-\overline{x}\rangle \\= & {} \Vert x_{n+1}{-}x_n\Vert ^2{+}\varphi _n-\varphi _{n+1} {+}\alpha (\varphi _n-\varphi _{n-1}){+}\alpha \Vert x_n{-}x_{n-1}\Vert ^2. \end{aligned}$$

Hence, by using the previous lemma, the required result holds. $\square$

The Opial Lemma that we recall below will play an important role in the proof of the main result of this paper.

### Proposition 10

(Opial Lemma) Let *H* be a real Hilbert space, $C\subseteq H$ a nonempty set and $\{x_n\}_{n=1}^\infty$ a given sequence such that:

(i) For every $z\in C, \lim _{n\rightarrow +\infty }\Vert x_n-z\Vert$ exists.
(ii) Every sequential weak cluster point of $\{x_n\}_{n=1}^\infty$ lies in *C*.

Then the sequence $\{x_n\}_{n=1}^\infty$ converges weakly to a point in *C*.

### Theorem 11

(i) The sequence $\{x_n\}_{n=1}^\infty$ converges weakly to a point in $\mathcal {S}$.
(ii) The sequence $(f(x_n))_{n=1}^\infty$ converges to the optimal objective value of the optimization problem (1).

### Proof

(i) According to Lemma 8, $\lim _{n\rightarrow +\infty }\Vert x_n-\overline{x}\Vert$ exists for all $\overline{x}\in \mathcal {S}$. Let *w* be a sequential weak cluster point of $\{x_n\}_{n=1}^\infty$. Then there exists a subsequence $\{x_{n_j}\}_{j=1}^\infty$ of $\{x_n\}_{n=1}^\infty$ such that $x_{n_j}$ converges weakly to *w* as $j \rightarrow +\infty$. By Lemma 8, we have that $w\in \mathrm{argmin}\,g$. This means that in order to come to the conclusion it suffices to show that $f(w)\le f(x)$ for all $x\in \mathrm{argmin}\,g$. From Lemma 9, Lemma 8 and the fact that $\sum _{n=1}^\infty \lambda _n=+\infty$, it follows that $\lim _{n\rightarrow \infty }[\Omega _n(x_n)-f(\overline{x})]\le 0$ for all $\overline{x}\in \mathcal {S}$. Thus,

$$\begin{aligned} f(w)\le \liminf _{j\rightarrow +\infty }f(x_{n_j})\le \lim _{n\rightarrow +\infty }\Omega _n(x_n)\le f(\overline{x}) \quad \forall \overline{x}\in \mathcal {S}, \end{aligned}$$

which shows that $w \in \mathcal {S}$. Hence, thanks to Opial Lemma, $\{x_n\}_{n=1}^\infty$ converges weakly to a point in $\mathcal {S}$.

(ii) The statement follows easily from the above considerations. $\square$

In the end of this section we present some situations where Assumption 2 is verified.

### Remark 12

Let $\alpha \in (0,1), c\in (1,+\infty ), q\in (0,1)$ and $\gamma \in \left( 0,\frac{2}{L_g}\right)$ be arbitrarily chosen. We set

$$\begin{aligned} K:= & {} \frac{2}{\alpha }>0,\\ \beta _n:= & {} \frac{\gamma [L_f+2((1+\alpha )K+c)]}{2-\gamma L_g}+(1-\alpha )\gamma Kn^q, \end{aligned}$$

and

$$\begin{aligned} \lambda _n:=\frac{(1-\alpha )\gamma }{\beta _n}, \end{aligned}$$

for all $$n\ge 1$$.

(i) Since $\beta _n\ge \frac{\gamma [L_f+2((1+\alpha )K+c)]}{2-\gamma L_g}$, we have $\beta _n(2-\gamma L_g)\ge \gamma [L_f+2((1+\alpha )K+c)]$, which implies that $\frac{L_n}{2}+\frac{\alpha -1}{\lambda _n}\le -\left( c+(1+\alpha )K\right)$ for all $n \ge 1$.
(ii) For all $n\ge 1$ it holds
  $$\begin{aligned} \beta _{n+1}-\beta _n=(1-\alpha )\gamma K[(n+1)^q-n^q]\le (1-\alpha )\gamma K=K\lambda _{n+1}\beta _{n+1}. \end{aligned}$$
(iii) It holds $\liminf _{n\rightarrow +\infty }\lambda _n\beta _n=\liminf _{n\rightarrow +\infty }(1-\alpha )\gamma >0$.
(iv) For all $n\ge 1$ we have
  $$\begin{aligned} \frac{1}{\lambda _{n+1}}-\frac{1}{\lambda _n}=\frac{1}{(1-\alpha )\gamma }\left( \beta _{n+1}-\beta _n\right) =K\left( (n+1)^q-n^q\right) \le K=\frac{2}{\alpha }. \end{aligned}$$
(v) Since $q\in (0,1)$, we have $\sum _{n=1}^\infty \frac{1}{\beta _n}=+\infty$, which implies that $\sum _{n=1}^\infty \lambda _n=+\infty$.
(vi) Finally, as $g\le \delta _{\mathrm{argmin}\,g}$, we have $g^*\ge (\delta _{\mathrm{argmin}\,g})^*=\sigma _{\mathrm{argmin}\,g}$ and this implies that $g^*-\sigma _{\mathrm{argmin}\,g}\ge 0$. We present a situation where Assumption 2(III) holds and refer to [10] for further examples. For instance, if $g(x)\ge \frac{a}{2}\mathrm {dist}^2(x,\mathrm{argmin}\,g)$ where $a>0$, then $g^*(x)-\sigma _{\mathrm{argmin}\,g}(x)\le \frac{1}{2a}\Vert x\Vert ^2$ for every $x \in H$. Thus, for $p\in \mathrm {ran}(N_{\mathrm{argmin}\,g})$, we have
  $$\begin{aligned} \lambda _n\beta _n\left[ g^*\left( \frac{p}{\beta _n}\right) -\sigma _{\mathrm{argmin}\,g}\left( \frac{p}{\beta _n}\right) \right] \le \frac{\lambda _n}{2a\beta _n}\Vert p\Vert ^2. \end{aligned}$$
  Hence $\sum _{n=1}^\infty \lambda _n\beta _n\left[ g^*\left( \frac{p}{\beta _n}\right) -\sigma _{\mathrm{argmin}\,g}\left( \frac{p}{\beta _n}\right) \right]$ converges, if $\sum _{n=1}^\infty \frac{\lambda _n}{\beta _n}$ converges or, equivalently, if $\sum _{n=1}^\infty \frac{1}{\beta _n^2}$ converges. This holds for the above choices of $\{\beta _n\}_{n=1}^\infty$ and $\{\lambda _n\}_{n=1}^\infty$ when $q\in \left( \frac{1}{2},1\right)$.

## Numerical example: image classification via support vector machines

In this section we employ the algorithm proposed in this paper in the context of image classification via support vector machines.

Having a set of training data $a_i \in \mathbb {R}^n, \ i=1, \ldots , k,$ belonging to one of two given classes denoted by “$-1$” and “$+1$”, the aim is to construct by using this information a decision function given in the form of a separating hyperplane, which assigns every new data to one of the two classes with a misclassification rate as low as possible. In order to be able to handle the situation when a full separation is not possible, we make use of non-negative slack variables $\xi _i \ge 0, \ i=1, \ldots , k$; thus the goal will be to find $(s,r, \xi ) \in \mathbb {R}^n \times \mathbb {R}\times \mathbb {R}^k_+$ as optimal solution of the following optimization problem

$$\begin{aligned} \begin{array}{rl} \text {minimize }\quad &{} \frac{1}{2}\Vert s\Vert ^2+\frac{C}{2}\Vert \xi \Vert ^2\\ \text {subject to }\quad &{} d_i(a_i^\top s+r)\ge 1-\xi _i, \quad \forall i=1,\ldots ,k\\ &{} \xi _i\ge 0, \quad \forall i=1,\ldots ,k, \end{array} \end{aligned}$$

where for $i=1,\ldots ,k, d_i$ is equal to $-1$ if $a_i$ belongs to the class “$-1$” and it is equal to $+1$, otherwise. Each new data $a \in \mathbb {R}^n$ will by assigned to one of the two classes by means of the resulting decision function $z(a)=a^\top s + r$, namely, *a* will be assigned to the class “$-1$”, if $z(a) < 0$, and to the class “$+1$”, otherwise. For more theoretical insights in support vector machines we refer the reader to [29].

By making use of the matrix

$$\begin{aligned} \mathbf {A} = \begin{bmatrix} d_1a_1^\top&\quad d_1&\quad 1&\quad 0&\quad \cdots&\quad 0 \\ d_2a_2^\top&\quad d_2&\quad 0&\quad 1&\quad \cdots&\quad 0 \\ \vdots&\quad \vdots&\quad \vdots&\quad \vdots&\quad \ddots&\quad \vdots \\ d_ka_k^\top&\quad d_k&\quad 0&\quad 0&\quad \cdots&\quad 1 \\ \mathbf {0}^\top _{\mathbb {R}^n}&\quad 0&\quad 1&\quad 0&\quad \cdots&\quad 0 \\ \mathbf {0}^\top _{\mathbb {R}^n}&\quad 0&\quad 0&\quad 1&\quad \cdots&\quad 0 \\ \vdots&\quad \vdots&\quad \vdots&\quad \vdots&\quad \ddots&\quad \vdots \\ \mathbf {0}^\top _{\mathbb {R}^n}&\quad 0&\quad 0&\quad 0&\quad \cdots&\quad 1 \\ \end{bmatrix} \in \mathbb {R}^{2k\times (n+1+k)} \end{aligned}$$

the problem under investigation can be written as

$$\begin{aligned} \begin{array}{rl} \text {minimize }\quad &{} \frac{1}{2}\Vert s\Vert ^2+\frac{C}{2}\Vert \xi \Vert ^2\\ \text {subject to }\quad &{} \mathbf {A}\left( \begin{array}{c} s\\ r\\ \xi \end{array}\right) -\left( \begin{array}{c} \mathbf {1}_{\mathbb {R}^k}\\ \mathbf {0}_{\mathbb {R}^k}\\ \end{array}\right) \in \mathbb {R}_+^{2k} \end{array} \end{aligned}$$

or, equivalently,

$$\begin{aligned} \begin{array}{rl} \text {minimize }\quad &{} \frac{1}{2}\Vert s\Vert ^2+\frac{C}{2}\Vert \xi \Vert ^2\\ \text {subject to }\quad &{} \left( \begin{array}{c} s\\ r\\ \xi \end{array}\right) \in \arg \min \frac{1}{2}\mathrm {dist}^2\left( \mathbf {A}(\cdot ) -\left( \begin{array}{c} \mathbf {1}_{\mathbb {R}^k}\\ \mathbf {0}_{\mathbb {R}^k}\\ \end{array}\right) ,\mathbb {R}_+^{2k}\right) . \end{array} \end{aligned}$$

By considering $f:\mathbb {R}^n\times \mathbb {R}\times \mathbb {R}^k\rightarrow \mathbb {R}$ as $$f\left( \begin{array}{c} s\\ r\\ \xi \end{array}\right) :=\frac{1}{2}\Vert s\Vert ^2+\frac{C}{2}\Vert \xi \Vert ^2$$, we have $$\nabla f\left( \begin{array}{c} s\\ r\\ \xi \end{array}\right) =\left( \begin{array}{c} s\\ 0\\ C\xi \end{array}\right) $$ and notice that $\nabla f$ is $\max \{1,C\}$-Lipschitz continuous.

Further, for $$g\left( \begin{array}{c} s\\ r\\ \xi \end{array}\right) :=\frac{1}{2}\mathrm {dist}^2\left( \mathbf {A}\left( \begin{array}{c} s\\ r\\ \xi \end{array}\right) -\left( \begin{array}{c} \mathbf {1}_{\mathbb {R}^k}\\ \mathbf {0}_{\mathbb {R}^k}\\ \end{array}\right) ,\mathbb {R}_+^{2k}\right) $$, we have $$\nabla g\left( \begin{array}{l} s\\ r\\ \xi \end{array}\right) =\mathbf {A}^\top \left( I-\mathrm{proj}_{\mathbb {R}^{2k}_{+}}\right) \left( \mathbf {A}\left( \begin{array}{c} s\\ r\\ \xi \end{array}\right) -\left( \begin{array}{c} \mathbf {1}_{\mathbb {R}^k}\\ \mathbf {0}_{\mathbb {R}^k}\\ \end{array}\right) \right) $$ and notice that $\nabla g$ is $\Vert \mathbf {A}\Vert ^2$-Lipschitz continuous, where $\mathrm{proj}_{\mathbb {R}^{2k}_{+}}$ denotes the projection operator on the set $\mathbb {R}^{2k}_{+}$.

**Fig. 1:**
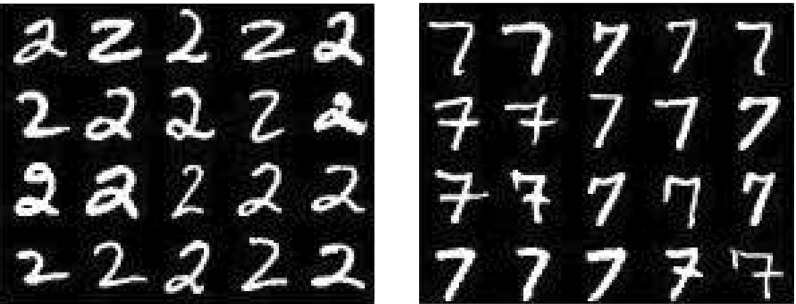
A sample of images belonging to the classes $-1$ and $+1$, respectively

For the numerical experiments we used a data set consisting of 6.000 training images and 2.060 test images of size $28\times 28$ taken from the website http://www.cs.nyu.edu/~roweis/data.html representing the handwritten digits 2 and 7, labeled by $-1$ and $+1$, respectively (see Fig. 1). We evaluated the quality of the resulting decision function on test data set by computing the percentage of misclassified images.

**Fig. 2:**
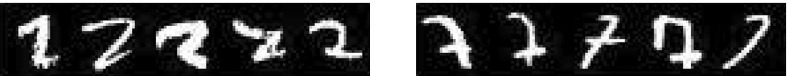
A sample of misclassified images

We denote by $\mathcal {D}=\{(X_i, Y_i), i=1,\ldots ,6.000\}\subset \mathbb {R}^{784}\times \{-1,+1\}$ the set of available training data consisting of 3.000 images in the class $-1$ and 3.000 images in the class $+1$. Due to numerical reasons each image has been vectorized and normalized. We tested in MATLAB different combinations of parameters chosen as in Remark 12 by running the algorithm for 3.000 iterations. A sample of misclassified images is shown in Fig. 2.

**Table 1:** Misclassification rate in percentage for different choices for the parameters $\alpha$ and *C* when $c=2$ and $q=0.9$

$\alpha$	$C=0.1$	$C=1$	$C=2$	$C=5$	$C=10$	$C=100$
0.1	2.2330	2.2330	2.2330	2.1845	2.1845	2.1845
0.3	2.2330	2.2816	2.2816	2.2816	2.2816	2.2816
0.5	2.2330	2.2330	2.2330	2.2816	2.2816	2.3301
0.7	2.3786	2.3786	2.3786	2.3786	2.3786	2.3786
0.9	2.9126	2.9126	2.9126	2.9126	2.8641	2.8155
0 ($\hbox {K}=0.1$)	3.1068	3.0583	3.0583	2.9612	2.9612	2.7184
0 ($\hbox {K}=1$)	2.2816	2.2330	2.2330	2.2330	2.2330	2.2330
0 ($\hbox {K}=10$)	2.2816	2.2330	2.2330	2.2330	2.2330	2.2330
0 ($\hbox {K}=100$)	2.2330	2.2330	2.2330	2.2330	2.2330	2.2330
0 ($\hbox {K}=1000$)	2.2330	2.2330	2.2330	2.2330	2.2330	2.2330

In Table 1 we present the misclassification rate in percentage for different choices for the parameters $\alpha \in (0,1)$ (we recall that in this case we take $K:=2/\alpha$) and $C>0$, while for $\alpha =0$ which corresponds to the noninertial version of the algorithm we consider different choices of the parameter $K>0$ and $C>0$. We observe that when combining $\alpha =0.1$ with each regularization parameters $C=5, 10, 100$ leads to the lowest misclassification rate with 2.1845%.

**Table 2:** Misclassification rate in percentage for different choices for the parameters *C* and $c>1$ when $\alpha =0.1$ and $q=0.9$

*C*	$c=1.1$	$c=2$	$c=5$	$c=10$	$c=100$
0.1	2.2330	2.2330	2.2330	2.2330	2.2330
1	2.2330	2.2330	2.2330	2.2330	2.2330
2	2.2330	2.2330	2.2330	2.2330	2.2330
5	2.1845	2.1845	2.1845	2.1845	2.1845
10	2.1845	2.1845	2.1845	2.1845	2.1845
100	2.1845	2.1845	2.1845	2.1845	2.1845

In Table 2 we present the misclassification rate in percentage for different choices of the parameters $C>0$ and $c>1$. The lowest classification rate of $2.1845\%$ is obtained for each regularization parameter $C=5, 10, 100$.

**Table 3:** Misclassification rate in percentage for different choices for the parameters *C* and $q\in (1/2,1)$ when $\alpha =0.1$ and $c=2$

*C*	$q=0.6$	$q=0.75$	$q=0.9$
0.1	2.2816	2.3301	2.2330
1	2.2330	2.2816	2.2330
2	2.2816	2.2816	2.2330
5	2.2330	2.2816	2.1845
10	2.2330	2.2816	2.1845
100	2.2330	2.2330	2.1845

Finally, Table 3 shows the misclassification rate in percentage for different choices for the parameters $C>0$ and $q\in (1/2,1)$. The lowest classification rate of $2.1845\%$ is obtained when combining the value $q=0.9$ with each regularization parameter $C=5, 10, 100$.

## References

[1] Alvarez F (2000). On the minimizing property of a second order dissipative system in Hilbert spaces. SIAM J. Control Optim..

[2] Alvarez F (2004). Weak convergence of a relaxed and inertial hybrid projection-proximal point algorithm for maximal monotone operators in Hilbert space. SIAM J. Optim..

[3] Alvarez F, Attouch H (2001). An inertial proximal method for maximal monotone operators via discretization of a nonlinear oscillator with damping. Set-Valued Anal..

[4] Attouch, H., Cabot, A., Czarnecki, M.-O.: Asymptotic behavior of nonautonomous monotone and subgradient evolution equations. Trans. Am. Math. Soc. (to appear) (2016). arXiv:1601.00767

[5] Attouch H, Czarnecki M-O (2010). Asymptotic behavior of coupled dynamical systems with multiscale aspects. J. Differ. Equ..

[6] Attouch H, Czarnecki M-O (2017). Asymptotic behavior of gradient-like dynamical systems involving inertia and multiscale aspects. J. Differ. Equ..

[7] Attouch H, Czarnecki M-O, Peypouquet J (2011). Prox-penalization and splitting methods for constrained variational problems. SIAM J. Optim..

[8] Attouch H, Czarnecki M-O, Peypouquet J (2011). Coupling forward-backward with penalty schemes and parallel splitting for constrained variational inequalities. SIAM J. Optim..

[9] Attouch H, Peypouquet J, Redont P (2014). A dynamical approach to an inertial forward-backward algorithm for convex minimization. SIAM J. Optim..

[10] Banert S, Boţ RI (2015). Backward penalty schemes for monotone inclusion problems. J. Optim. Theory Appl..

[11] Bauschke HH, Combettes PL (2011). Convex Analysis and Monotone Operator Theory in Hilbert Spaces. CMS Books in Mathematics.

[12] Bertsekas DP (1999). Nonlinear Programming.

[13] Boţ RI, Csetnek ER (2014). Forward-backward and Tseng’s type penalty schemes for monotone inclusion problems. Set-Valued Var. Anal..

[14] Boţ RI, Csetnek ER (2014). A Tseng’s type penalty scheme for solving inclusion problems involving linearly composed and parallel-sum type monotone operators. Vietnam J. Math..

[15] Boţ, R.I., Csetnek, E.R.: Levenberg–Marquardt dynamics associated to variational inequalities. Set-Valued Var. Anal. (2017). doi:10.1007/s11228-017-0409-8

[16] Boţ RI, Csetnek ER (2016). An inertial forward-backward-forward primal-dual splitting algorithm for solving monotone inclusion problems. Numer. Algorithms.

[17] Boţ RI, Csetnek ER (2016). An inertial alternating direction method of multipliers. Minimax Theory Appl..

[18] Boţ RI, Csetnek ER (2015). A hybrid proximal-extragradient algorithm with inertial effects. Numer. Funct. Anal. Optim..

[19] Boţ RI, Csetnek ER (2016). An inertial Tseng’s type proximal algorithm for nonsmooth and nonconvex optimization problems. J. Optim. Theory Appl..

[20] Boţ RI, Csetnek ER (2016). Approaching the solving of constrained variational inequalities via penalty term-based dynamical systems. J. Math. Anal. Appl..

[21] Boţ RI, Csetnek ER (2017). Penalty schemes with inertial effects for monotone inclusion problems. Optimization.

[22] Boţ RI, Csetnek ER (2017). Second order dynamical systems associated to variational inequalities. Appl. Anal..

[23] Boţ, R.I., Csetnek, E.R.: A second order dynamical system with Hessian-driven damping and penalty term associated to variational inequalities (2016). arXiv:1608.04137 10.1080/02331934.2018.1452922 PMC6817320 31708645

[24] Boţ RI, Csetnek ER, Hendrich C (2015). Inertial Douglas–Rachford splitting for monotone inclusion problems. Appl. Math. Comput..

[25] Boţ RI, Csetnek ER, László S (2016). An inertial forward-backward algorithm for the minimization of the sum of two nonconvex functions. EURO J. Comput. Optim..

[26] Cabot A, Frankel P (2011). Asymptotics for some proximal-like method involving inertia and memory aspects. Set-Valued Var. Anal..

[27] Chen C, Chan RH, MA S, Yang J (2015). Inertial proximal ADMM for linearly constrained separable convex optimization. SIAM J. Imaging Sci..

[28] Chen C, MA S, Yang J (2015). A general inertial proximal point algorithm for mixed variational inequality problem. SIAM J. Optim..

[29] Cristianini N, Taylor JS (2000). An Introduction to Support Vector Machines and Other Kernel-Based Learning Methods.

[30] Maingé P-E (2008). Convergence theorems for inertial KM-type algorithms. J. Comput. Appl. Math..

[31] Maingé P-E, Moudafi A (2008). Convergence of new inertial proximal methods for dc programming. SIAM J. Optim..

[32] Moudafi A, Oliny M (2003). Convergence of a splitting inertial proximal method for monotone operators. J. Comput. Appl. Math..

[33] Noun N, Peypouquet J (2013). Forward-backward penalty scheme for constrained convex minimization without inf-compactness. J. Optim. Theory Appl..

[34] Ochs P, Chen Y, Brox T, Pock T (2014). iPiano: Inertial proximal algorithm for non-convex optimization. SIAM J. Imaging Sci..

[35] Peypouquet J (2012). Coupling the gradient method with a general exterior penalization scheme for convex minimization. J. Optim. Theory Appl..

[36] Polyak BT (1987). Introduction to Optimization, (Translated from the Russian) Translations Series in Mathematics and Engineering.

